# Mapping the distribution of *Anopheles funestus* across Benin highlights a sharp contrast of susceptibility to insecticides and infection rate to *Plasmodium* between southern and northern populations

**DOI:** 10.12688/wellcomeopenres.10213.2

**Published:** 2017-03-03

**Authors:** Rousseau Djouaka, Romaric Akoton, Genevieve M. Tchigossou, Seun M. Atoyebi, Helen Irving, Michael O. Kusimo, Innocent Djegbe, Jacob M. Riveron, Eric Tossou, Akadiri Yessoufou, Charles S. Wondji

**Affiliations:** 1 International Institute of Tropical Agriculture, Cotonou, Benin; 2 University of Abomey-Calavi, Cotonou, Benin; 3 Cell Biology and Genetics Unit, Department of Zoology, University of Ibadan, Ibadan, Nigeria; 4 Liverpool School of Tropical Medicine, Liverpool, UK; 5 University of Sciences, Arts and Techniques of Natitingou, Ecole Normale Supérieure de Natitingou, Natitingou, Benin

**Keywords:** Anopheles funestus, distribution, Plasmodium infection, insecticide resistance, South-North, Benin

## Abstract

**Background. **Malaria remains an important public health issue in Benin, with *Anopheles gambiae s.l.* and *Anopheles funestus s.s* being the predominant vectors. This study was designed to generate information on *An. funestus* distribution, molecular speciation, *Plasmodium* infection rate and insecticide susceptibility status across Benin. **Methods. **Mosquito samples were collected from December 2014 to January 2016 in 46 localities in Benin. These samples were mapped and *An. funestus *collected were speciated to the molecular level. *Plasmodium* infection rate was determined using a Taqman assay and susceptibility to insecticides was assessed using the WHO guidelines. The genotyping of the L119F- Gste2 mutation was also carried out. **Results.** *An. funestus* was found in 8 out of the 46 localities surveyed with a high presence in Tanongou (wet Sudanese ecological zone), Kpome, Doukonta and Pahou (sub-equatorial ecological zone). Molecular identifications revealed that only *An. funestus* *s.s* was present in southern Benin, whereas in Tanongou (northern Benin) *An. funestus s.s.* and *An. leesoni* were found in sympatry at proportions of 77.7% and 22.3% respectively. *Plasmodium* infection rate of *An. funestus* was higher in southern Benin at a range of 13 to 18% compared to 5.6% recorded in Tanongou. High DDT (8±0.5%) and permethrin (11±0.5%) resistance were observed in Doukonta, Kpome and Pahou, contrasting with relatively low resistance profiles: mortality-DDT=90±3.18% and mortality-permethrin=100% in Tanongou. Genotyping analysis revealed  high frequency  of the resistant 119F allele in the South (Kpome and Doukonta) compared to the North (Tanongou). **Discussion and Conclusion. **The high presence of *An. funestus* in the South compared to the North  could be due to favorable environmental and climatic conditions found in both regions. A significant *Plasmodium* infection rate was recorded across the country. A high resistance profile was recorded in the southern Benin; this raises the need for further investigations on resistance selection factors.

## Background

Malaria remains a major public health challenge in Benin, with the most vulnerable populations being children less than five years and pregnant women ^1^. It accounts for around 37% of hospital consultations in the country ^2^. Efforts to eradicate this disease in Africa have focused on treatment of diagnosed cases and preventive strategies, which are mainly based on vector control, such as the use of insecticide treated nets, indoor residual spraying of insecticides and larviciding ^1^.

In the past decade, vector control interventions have massively contributed to the significant decrease observed in the burden of malaria across Africa, notably in Benin ^3^. To sustain such progress, national control programs need better knowledge on key malaria vectors nationwide, including their geographical distribution, susceptibility profile to insecticides and contribution to malaria transmission, as well as understanding the vectorial complexity of these species. Such information already exists for *Anopheles gambiae* across Benin ^2, 4, 5^, but this is not the case for the other major vector *An. funestus,* for which only limited information is available, mainly from few coastal populations ^6, 7^

*An. funestus* Giles is one of the key malaria-transmitting mosquitoes in Africa. The vectorial capacity of this mosquito vector is close to and could exceed that of *An. gambiae*, the most documented malaria vector in some countries ^8^. *An. funestus* Giles group is made up of nine species distributed across sub-Saharan Africa ^9, 10^. These nine species of the *An. funestus* group are as follows: *An. funestus* Giles (s.s), *An. vaneedeni* Gillies and Coetzee *, An. leesoni* Evans, *An. parensis* Gillies, *An. rivulorum* Leeson *, An. fuscivenosus* Leeson, *An.* *brucei* Service, *An. aruni* Sobti and *An. confusus* Evans and Leeson. These species are not easily distinguishable using morphological keys ^9, 10^.

The vectorial capacity of members of the *An. funestus* group varies significantly, with most species being zoophilic, except *An. funestus s.s.,* which is the main *Plasmodium* vector in this group. Indeed high infection rates have been reported for *An. funestus s.s*., such as 22% ^11^ and 27% ^12^ documented in South Africa, 11% in Tanzania ^13^, 50% in Burkina Faso ^14^ and 18% in Benin ^7^. However, other members of the group, such as *An. rivulorum* has a high anthropophilic rate of 40% (42/106) in the southern region of Nigeria ^15^, but presents a low contribution to malaria transmission in Tanzania ^16^. As for *An. vaneedeni,* this species could be either exophilic or anthrophilic, but can easily carry the *Plasmodium* parasite under laboratory conditions ^17^, whereas *An. parensis* is endophilic, but does not carry the malaria parasite ^15, 18, 19^. In most parts of Africa, *An. funestus s.s.* and other members of the *An. funestus* group live in sympatry ** ^9, 15, 18^, and if appropriate identification is not made this could lead to wrong vectorial characterization of *An. funestus s.s*. This relevant information on *An. funestus* in Benin has been documented in some parts of the southern coastal localities of Ouidah, Kpomasse, Tori and Pahou ^6, 20^, but no extensive study has so far been carried out in a North-South Benin transect to determine the extent of the distribution of this species in the country and its contribution to malaria transmission.

The resistance profile of *An. funestus s.s* has only been explored for some coastal populations with a multiple resistance to pyrethroids, DDT and carbamates reported in the locations of Pahou ^6^ and Kpome ^7^. It remains to be established whether such resistance is distributed nationwide or not. The resistance of *An. funestus* species to several insecticides used in public health has been well documented in many other African countries, and for some the resistance pattern and underlying resistance mechanisms have been the same nationwide, for example in Uganda ^19^, whereas variations have also been observed, such as in Malawi ^21^. Across Africa, ** the ** resistance profile of *An. funestus s.s.* significantly varies with resistance to pyrethroids and carbamates observed in southern Africa (Mozambique, Malawi and South-Africa) ^22– 25^, whereas East African (Uganda and Kenya) populations of *An. funestus* are resistant to pyrethroids and DDT, but susceptible to carbamates ^19, 26^. Central (Cameroon) ^27, 28^ and West African (Ghana, Benin) populations are resistant to pyrethroid, organochlorines and carbamates ^6, 29^. In Benin, *An. funestus s.s.* population in the coastal locality of Pahou is resistant to pyrethroids, carbamates and is highly resistant to DDT ^6^. Furthermore, ** it was demonstrated that the GSTe2 gene with the L119F mutation accounts for its capacity to metabolize DDT ^30^.

This study aims to generate information on the distribution, *Plasmodium* infection rate and resistance status of *An. funestus* in the South-North transect of Benin to help control programs to have a better assessment of the contribution of this species nationwide and how best to control it.

## Methods

### Ethical statement

No ethical permit was required for this study. However, there was a focus group discussion with the community and household heads where verbal consent was obtained for mosquito collections in the community after the study aims and objectives were explained. During this research study, we did not perform insecticide spraying, night collections, or human bait for mosquito collection. All mosquitoes were sampled during daytime using electrical aspirators activated with batteries.

### Study sites and mosquito collection

**Figure 1.**
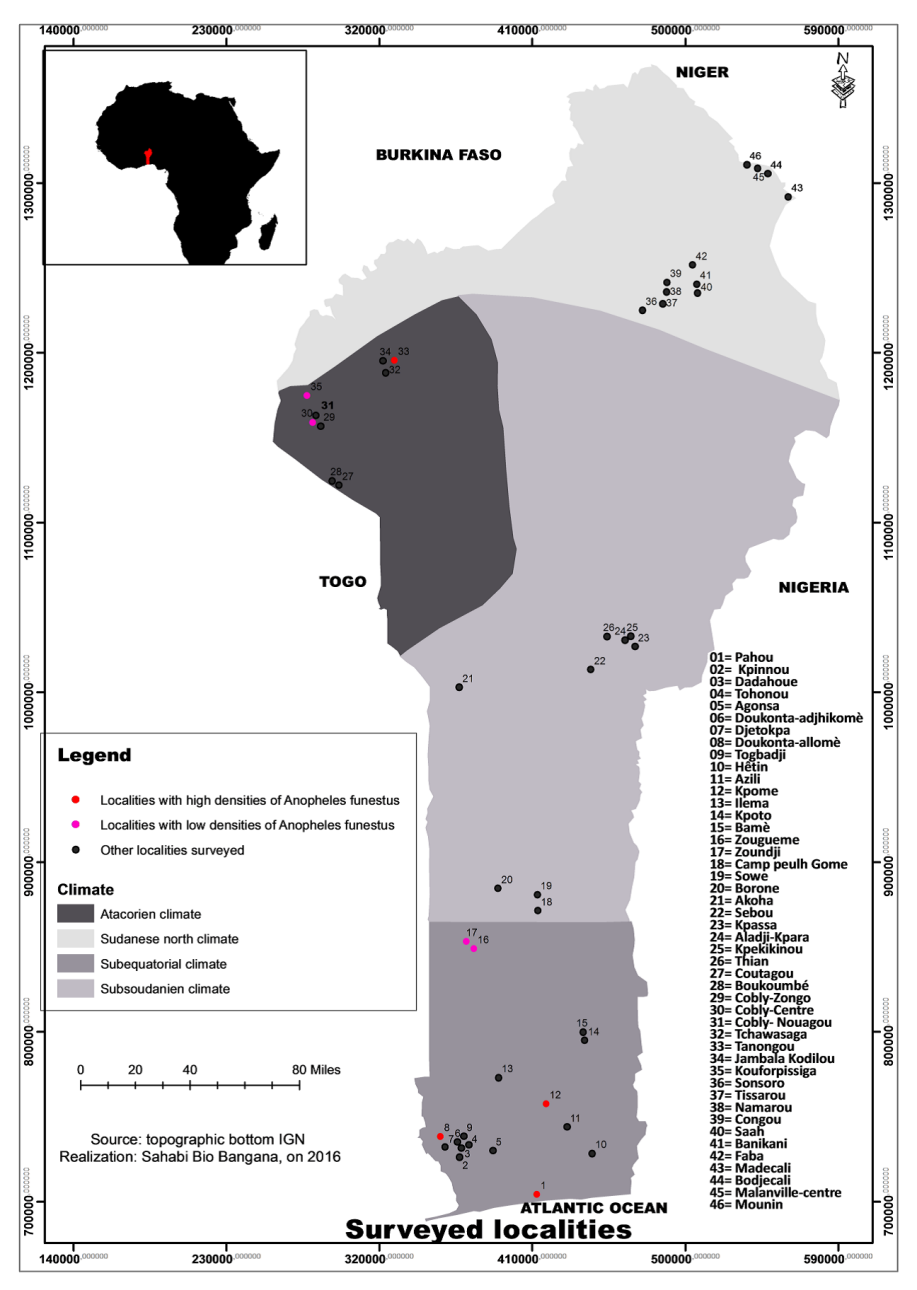
Surveyed localities between December 2014 and January 2016 in South-North of Benin.

***Study site description.*** Benin lies between the Equator and the Tropic of Cancer at latitudes ranging from 6°30′ N to 12°30′ N and longitude from 1° E to 3°40′ E. This country shares boundaries with Togo in the West, Burkina Faso and Niger in the North, and Nigeria in the East. Four main climatic zones are found in the country. The North Sudanese climatic region, which is characterized by one long dry season and a short rainy season, with low relative humidity and rainfall that is the lowest in the country (800 to 1000 mm per year). Large water bodies are found in this region and temperatures are the highest, and could reach 45°C during dry seasons. The second region is the wet Sudanese climatic zone (Atacorian). This climatic region is dominated by hills of up to 800 m of altitude and several small water bodies, which makes the region colder. Annual rainfall ranges from 1200 to 1300 mm per year, the vegetation is partially of wet savanna type, the temperature in this part of the country is the lowest. The third region is the sub-Sudanese climatic region that covers the center of the country and part of the South. This climatic region has one long rainy season and one short dry season. Rainfall is between 900 and 1200 mm, the region is less hilly and the vegetation is of wet savanna type. The fourth region is the southern sub-equatorial climatic region that spans the southern part of the country and extends up to coastal areas of Benin. This region is made up of two rainy seasons and two dry seasons. The relative humidity is high, temperatures are relatively low and the vegetation is a mosaic of coastal, wetlands, forest, and wet savanna type. Several water bodies join together in this part of the country before being channeled into the sea ( Figure 1).

***Mosquito sampling.*** From December 2014 to January 2016, indoor collections of adult female mosquito were made between 06 to 10am in several localities along South-North transect of Benin using four electric aspirators. Mosquito collections were carried out in different localities and the GPS was used to determine the latitude and longitude for each sampled locality. Maps of surveyed sites and the distribution of *An. funestus* in Benin were developed using recorded latitudes and longitudes. For each surveyed locality, a minimum of 30 rooms were randomly selected for mosquito aspirations. These rooms were selected in a way to cover the various ecologies found in each locality. At least three days were spent in each surveyed site but for localities where *An. funestus* were found, the number of days was extended to five days to obtain a good number of mosquitoes to be used for various analyses. Aspirated mosquitoes were identified morphologically ^9^, counted and the total number for each species was recorded. All blood-fed and gravid *An. funestus* (F _0_) collected inside houses were taken to the IITA insectary in Cotonou (Benin), where they were kept in small cups until fully gravid. The forced egg laying technique described by Morgan *et al.* ^26^ was then used to induce female *An. funestus* to lay eggs. Egg batches and emerging larvae from the same female mosquito were reared together and later pooled with larvae from other females, if these females were found belonging to the same molecular species. The insectary condition was at a temperature and relative humidity of 25–27°C and 80±5% respectively. Larvae were fed daily with Tetramin™ baby fish food and the water of each larvae bowl was changed every two days to reduce the mortality. The F _1_ adults generated were randomly mixed in cages for subsequent experiments.

### Seasonal estimation of mosquito densities per room

Mosquito densities per room (m/r) were estimated during four annual climatic seasons: rainy season, transition from rainy to dry season, dry season and transition from dry to rainy season. This estimation was based on the total number of *An. funestus s.l.* collected during each season divided by the number of rooms surveyed for mosquito collections in that season. Seasonal variations of *An. funestus* densities were determined per room in two localities in Benin: the locality of Tanongou in the North (wet Sudanese/Atacorian climatic region) and the locality of Kpome in the South (subequatorial climatic region). Kpome and Tanongou were selected to represent the southern and northern regions respectively, due to the high density of *An. funestus* recorded in these localities.

### PCR species identification

For each locality, female mosquito specimens that were morphologically identified as belonging to *An. funestus* group ^9^ were subjected to DNA extractions using Qiagen DNeasy Kit followed by PCR for species identification, as described by Koekemoer *et al.* ^31^.

### *Plasmodium* infection rate of *An. funestus* populations from surveyed localities

The *Plasmodium* infection rate was determined using the TaqMan assay ^32^. The reaction was performed in a 10µl final volume reaction containing 1×SensiMix (Bioline), 800 nM of each primer: PlasF, 5’-GCTTAGTTACGATTAATAGGAGTAGCTTG-3’ and PlasR, 5’- GAAAATCTAAGAATTTCACCTCTGACA-3’, and 200 nM of probes labeled with fluorophores: FAM (5'-TCTGAATACGAATGTC-3') for detecting *P. falciparum*, and HEX (5'-CTGAATACAAATGCC-3') for *P. ovale*, *P. vivax* and *P. malariae (P. ovm)*. *P. falciparum* sample and a mixture of *P. ovale, P. vivax* and *P. malariae* were used as positive controls. The real-time PCR Agilent MX 3005 system was used for amplification with the following cycling conditions: 95°C for 10 minutes for denaturation, followed by 40 cycles of 15 seconds at 92°C and 1 minute at 60°C.

### Insecticide susceptibility tests

Protocols and standard insecticide treated papers supplied by WHO ^33^ were used to test for insecticide susceptibility of *An. funestus* from selected localities in the northern and southern where there was a consistent number of ovipositing females. These selected localities were Tanongou, northern Benin in the wet Sudanese climatic region (Atacorian region), and Doukonta, southern Benin in the sub-equatorial climatic region. We assessed the susceptibility pattern of *An. funestus s.s.* from both localities to two insecticides of public health interest: pyrethroids type I permethrin (0.75%) used for insecticide treated nets (ITNs), and organochlorines DDT (4%) used in insecticide residual spraying (IRS). Exposed mosquitoes were fed with 10% sugar solution after 1hr of insecticide exposure after which mortalities were recorded 24hrs post exposure to insecticide treated papers ^33^. The wild population of *An. funestus* was exposed to non-treated insecticide papers as a control ^33^ due to lack of susceptible strains of *An. funestus*, ( *An. funestus* FANG). Prior to the experiment, the effectiveness of insecticide treated papers purchased from the Vector Biology Department, Liverpool School of Tropical Medicine (LSTM), UK was confirmed by exposing the susceptible strain *An. gambiae kisumu* to insecticide impregnated papers. Tests were conducted at a temperature and relative humidity of 25–27°C and 80±5% respectively. WHO criteria were used to determine resistance status with mortality between 98–100% indicating susceptibility, 90–97% potential resistance, and less than 90% resistance ^33^.

### Distribution of L119F-GSTe2 resistance allele using TaqMan assay

To assess the role of L119F mutation in DDT resistance, wild female *An. funestus s.s.* collected from each selected location were genotyped using the Taqman assay, as previously demonstrated ^30^. The reaction was performed in a 10μl final volume containing 1×SensiMix (Bioline, London, UK), 800 nM of each primer and 200 nM of each probe using an Agilent MX3005P machine. The following cycling conditions were used: 10 min at 95°C, 40 cycles of 15s at 92°C and 1 min at 60°C. Two probes labelled with fluorochromes FAM and HEX were used. The FAM was used to detect the mutant allele, while the HEX detected the wild type allele.

### Data analysis

MedCalc easy-to-use online statistical software ^34^ using the Fisher’s exact test was used to test for significant difference of *Plasmodium* infection rate and L119F-GSTe2 genotyping data in the South compared to the North of Benin.

## Results

### Distribution of *Anopheles funestus* species in a South-North transect of Benin

Out of the 46 surveyed localities ( Figure 1 and Supplementary Table 1) in this study, *An. funestus* species were found in eight localities, generally in sympatry with *An. gambiae,* and spread in two geo-climatic regions of Benin. In addition, most of the sites where *An. funestus* species were collected were found in the western part of the country (six out of eight localities with *An. funestus*; Figure 1).

A total of 3179 mosquitoes belonging to different species were caught during this survey. These mosquito populations from indoor collections were dominated by *Anopheles spp.* 82.89% (2635), followed by *Culex spp.* 14.90% (474), *Mansonia spp.* 1.38% (44) and *Aedes spp.* 0.81% (26) ( Supplementary Table 1). Out of the morphologically identified *Anopheles spp.*, *An. gambiae s.l.* constituted 79% (2083), followed by *An. funestus s.l.* with 21% (552). No other *Anopheles* species was collected during the sampling period.

### Distribution of *An. funestus* in various geo-climatic regions of Benin

*An. funestus* was not found in either the dry Sudanese climatic region (no *An. funestus* collected in the 11 surveyed localities), nor in the transition region between the Sudanese and the sub-equatorial climatic regions (the sub-Sudanese climatic region), where no *An. funestus* was found in the nine surveyed localities ( Figure 1 and Supplementary Table 1). All *An. funestus* samples collected were either from the southern sub-equatorial region ( *An. funestus* found in five out of the 17 surveyed localities) or the northern wet Sudanese climatic region of the Atacora (three localities with *An. funestus* out of nine surveyed). It is worth indicating that most sampled specimens of *An. funestus* were found in the western part of Benin (six localities out of eight with *An. funestus*). Out of the 552 morphologically identified *An. funestus* sampled during this survey, 319 samples were from localities situated in the sub-equatorial climatic region and 233 from the wet Sudanese climatic region (Atacorian region). High densities of *An. funestus* were recorded in Kpome (243 *An. funestus*) and Tanongou (229 *An. funestus*), localities from the sub-equatorial climatic region and the wet Sudanese climatic region, respectively ( Figure 1).

### Seasonal variations of *An. funestus* density in the northern (Tanongou) and the southern (Kpome) localities of Benin

**Figure 2.**
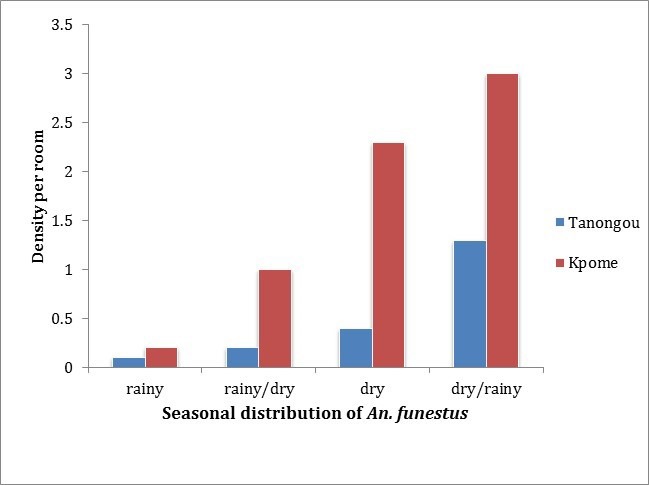
Seasonal distribution of *Anopheles funestus* (densities per room) in Kpome and Tanongou estimated from mosquito samples collected between December 2014 and January 2016.

Generated data from Kpome during the four monitored seasons revealed a higher *An. funestus* density per room (m/r) during the transition period from dry to rainy season (3 m/r). The lowest number of *An. funestus* (0.2 m/r) was recorded during rainy season. Densities of 1 and 2.3 m/r were documented during the transition from rainy to dry season and the dry season, respectively. A similar trend was observed in Tanongou with a higher density of *An. funestus* recorded during the transition from dry to rainy season (1.3 m/r), followed by the dry season with a density of 0.4 m/r, the transition from rainy to dry season and the rainy season had densities of 0.2 and 0.1 m/r, respectively. Comparative analysis of *An. funestus* densities at Kpome and Tanongou revealed a relatively higher rate of *An. funestus* mosquitoes per room at Kpome throughout all the four identified seasons compared to Tanongou ( Figure 2).

### Distribution of members of *An. funestus* group across Benin

PCR species detection of the 552 morphologically identified *An. funestus* individuals revealed a predominance of *An. funestus s.s.* in the two climatic regions where *An. funestus* was found in Benin. In the wet Sudanese climatic region, and more specifically in Tanongou, *An. funestus s.s.* was found in sympatry with its sister species *An. leesoni*. Out of the 229 *An. funestus s.l.* aspirated indoors at Tanongou, 178 were *An. funestus s.s.* and 51 were *An. leesoni*. In contrast, in the southern locality of Kpome where the highest density of *An. funestus* was recorded (243 *An. funestus s.l.*), as well as Doukonta and Pahou, no other member of the group apart from *An. funestus s.s.* was found ( Table 1).

**Table 1.** Distribution of members of *Anopheles funestus* group collected from December 2014 to January 2016 in the North-South Benin.

Localities	*An. funestus s.l.* subjected to molecular speciation	*An. funestus s.s.*	*An. leesoni*
Doukonta	15	15	0
Zoundji	3	3	0
Zougueme	1	1	0
Kouforpissiga	3	3	0
Cobly centre	1	1	0
Pahou	57	57	0
Tanongou	229	178	51
Kpome	243	243	0
Total	552	501	51

### *Plasmodium* infection rate of identified members of *An. funestus* group

Taqman results (n=552) showed that *An. funestus* mosquitoes from the sub-equatorial climatic localities of the southern Benin were significantly infected with *Plasmodium* compared with those from the wet Sudanese localities of the northwestern Benin (Atacorian region) (P=0.0001). *An. funestus* from Kpome, Pahou and Doukonta in southern Benin had *Plasmodium* infection rates of 18.51, 15.78 and 13.33%, respectively. However, in northwestern Benin, only *An. funestus s.s.* from Tanongou was infected with *Plasmodium* with an infection rate of 5.62% ( Table 2). *Plasmodium* infection was absent in all the 51 *An. leesoni* specimens analysed during this course of research ( Table 2).

**Table 2.** *Plasmodium* infection rate of members of *Anopheles funestus* group collected from December 2014 to January 2016 in different localities of Benin.

Locality	Species	Mosquito analyzed	Total infected	*Plasmodium* infection rate (%)
Kpome	*An. funestus s.s.*	243	45	18.51
Pahou	*An. funestus s.s.*	57	9	15,78
Doukonta	*An. funestus s.s.*	15	2	13.33
Cobly	*An. funestus s.s.*	1	0	0
Koufforpissiga	*An. funestus s.s.*	3	0	0
Zoundji	*An. funestus s.s.*	3	0	0
Zoungueme	*An. funestus s.s.*	1	0	0
Tanongou	*An. funestus s.s.*	178	10	5.62
	*An. leesoni*	51	0	0
	Total	552	66	

### Comparative insecticide susceptibility tests of *An. funestus s.s.* in the northern (Tanongou) and the southern (Doukonta) localities of Benin

**Figure 3.**
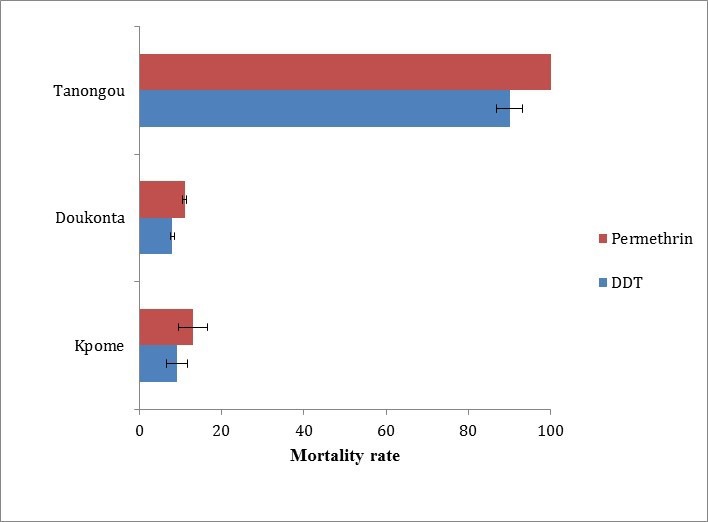
Insecticide resistance profiles of *Anopheles funestus* populations in Kpome (South Benin), Doukonta (South Benin) and Tanongou (North Benin). Error bar represents standard deviation of the mean.

Insecticide susceptibility tests of *An. funestus s.s.* from Doukonta, Pahou ^6^ and Kpome ^7^ in the South, and Tanongou in northern Benin were assessed. In total, 100 females each (F _1_ generated from F _0_ oviposition: 75 oviposited out of 110 and 9 oviposited out of 15 *An. funestus s.s.* from Tanongou and Doukonta respectively) of *An. funestus s.s.* from Doukonta were exposed to DDT and permethrin in 4 replicates (pools of 25 mosquitoes). Similarly, 100 *An. funestus s.s.* from Tanongou were exposed to permethrin and DDT in 5 replicates (average pool of 20 mosquitoes). Results revealed low mortalities to DDT (8±0.5%) and permethrin (11±0.5%) for *An. funestus s.s.* from Doukonta, whereas the Tanongou population had higher mortality rates to DDT (90±3.18%) and permethrin (100%). This shows that there is a higher resistance in Doukonta compared to Tanongou ( Figure 3). Similarly, high resistance levels have been previously documented in southern localities of Pahou and Kpome ^6, 7^.

### Screening of L119F- GSTe2 mutation in a wild population of *Anopheles funestus* from Benin

**Figure 4.**
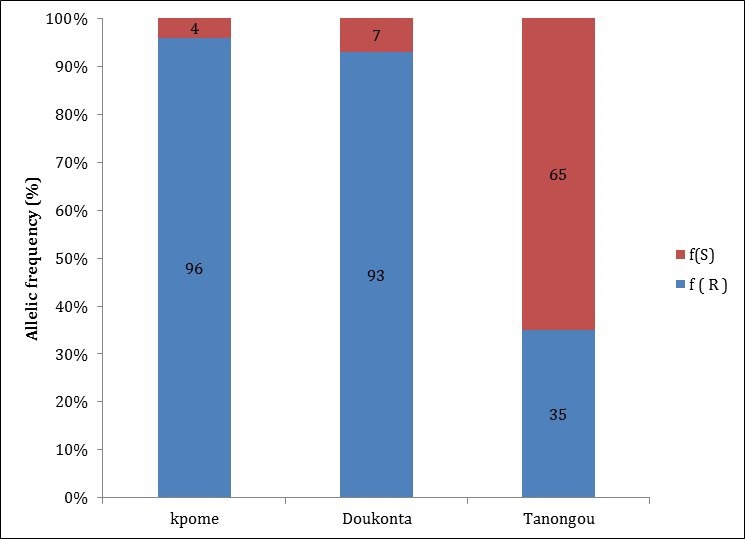
Allelic frequency of the L119F-GSTe2 mutation in wild *Anopheles funestus* populations (F _0_) from Kpome (South Benin), Doukonta (South Benin) and Tanongou (North Benin). f(S) represents frequency of the susceptible allele and f(R) represents the frequency of the resistant allele in the population.

Genotyping of the L119F-Gste2 mutation in wild *An. funestus* population from each of the selected locations revealed the presence of the resistant 119F allele at a high frequency: 96% in Kpome ^7^, 83.2% in Doukonta (southern Benin), while in Tanongou (North Benin), 35% mutant allelic frequency was recorded. No susceptible allele (SS) was observed either in Kpome or Doukonta mosquitoes, showing that the 119F gene is close to fixation in the *An. funestus* populations of these two locations in the southern Benin. A significant difference (P≤0.0001) was observed between the 119F allelic frequency recorded in Kpome and Doukonta, where a high resistance to DDT was observed compared to Tanongou ( Figure 4).

## Discussion

This research was designed to map the distribution of *An. funestus* in Benin and compare the insecticide resistance profile of this malaria vector in the North-South transect, as well as their infection rates with *Plasmodium* species, for improved knowledge on this malaria vector and enhanced performances of current malaria control tools.

### Distribution of *An. funestus* and its implication in malaria transmission in the various geo-climatic settings of Benin

*An. funestus* was mainly found in the southern and the northwestern localities of Benin in this study. In these two geo-climatic regions, there seem to be a high tendency of this species to colonize the western areas of the country (north and southwestern). The relatively high presence of this vector in the western part of Benin could be explained by the humidity, relatively low temperatures associated with the hilly landscape, and the presence of rivers and streams covered with vegetation ^35^. This shows that this species prefers more permanent water bodies with vegetation usually found along rivers, streams and lakes ^36^, whereas *An. gambiae* tends to oviposit in temporary breeding sites, such as puddles and animal foot prints ^37^. Very little or no population of *An. funestus* was found in the dry Sudanese climatic region of northeastern Benin. The low presence of this mosquito species in this dry hot region (low rain falls and temperature reaching 45°C during dry seasons) is either due to the period of sampling or the low presence of permanent fresh water bodies covered with vegetation coupled with dryness of the region ^38^.

The density of *An. funestus* species collected indoor in this research further confirms their endophilic behavior ^39^. Two species of the *An. funestus* group were identified during this study: *An. funestus s.s.* and *An. leesoni*. Contrary to *An. funestus s.s.,* there was no trace of *Plasmodium* DNA in the 51 samples of *An. leesoni* analyzed. This result confirms the low/no implication of *An. leesoni* in the transmission of malaria, as previously documented ^14, 39^, which is notable in West Africa as this species ** is known to be highly zoophilic. While placing a low epidemiological interest on *An. leesoni*, this study further highlights the need for a high focus on *An. funestus s.s.* for improved control of malaria in Benin ^7^. Recorded infection rates of *An. funestus* were more than three times higher in screened localities of southern localities (Kpome, Pahou, Doukonta) compared to the North (Tanongou), suggesting a higher implication of *An. funestus* in malaria transmission in the southern part of the country where its density is also high. The high *Plasmodium* infection rates observed in southern Benin are similar to some infection rates documented in several African countries in this species; *Plasmodium falciparum* infection rates of 22 ^11^ and 27% ^12^ have been found in *An. funestus* populations of South Africa. In countries from the western part of Africa, a mean rate of infectivity between 3 and 15% has been observed, including in Burkina Faso ^14, 40^ and recently in Ghana ^41^. In Burkina Faso, Dabire *et al.* ^40^ documented the presence of *Plasmodium* in *An. funestus* (20% infection rate) from Lena during the month of August 2000. In Benin, two studies recently conducted in southern localities revealed *Plasmodium* infection rates of 13.6 and 18.27% in *An. funestus* ^7, 42^. This study has shown a similar trend in the densities of *An. funestus* in both screened ecological zones throughout the year. High densities of *An. funestus* mosquitoes were recorded during the transition from dry to rainy season followed by the dry season, then the transition from the rainy to dry season and finally the rainy season, where the least density of *An. funestus* were recorded. The involvement of *An. funestus* in the transmission of malaria during dry seasons was also documented in Ghana ^43^, Nigeria ^15^, Burkina Faso ^14, 40^, and more recently in southern Benin ^44^.

### Comparative insecticides susceptibility tests of *An. funestus s.s.* from southern (Doukonta) and northern (Tanongou) localities of Benin

Comparative analysis of insecticide resistance profiles in *An. funestus* populations from Doukonta (southern Benin) and Tanongou (northern Benin) reveals that *An. funestus s.s.* from Doukonta are relatively more resistant to DDT and permethrin (mortality rates of 8±0.5 and 11±0.5%, respectively) than those from Tanongou, where only a moderate resistance was observed to DDT (mortality rate of 90±3.18%) and a full susceptibility to permethrin (100%). High resistance to DDT and permethrin had previously been reported in populations of *An. funestus* from two other localities of southern Benin, Pahou and Kpome ^6, 7^. In addition to the use of agricultural insecticides in both the northern and southern surveyed sites, the high insecticide resistance observed in the South could be associated with environmental factors, such as urbanization, which increases the level of xenobiotics (pollution) in *Anopheles* breeding sites and could favor the selection of cross resistance to permethrin and DDT in southern Benin compared to northwestern Benin with less urbanization and pollution ^45^. Recorded resistance profiles could also be associated with a relatively high flow of genes among *An. funestus* populations in southern Benin compared to the North, particularly if there are some barriers to gene flow, which needs to be investigated further. Other factors of resistance selection, such as the relatively high use of ITNs/IRS (use of public health insecticides) in the southern Benin compared to the North, might have also contributed to observed high resistance profile of mosquitoes ^6, 46– 50^. Similar observations have been documented on *An. gambiae s.l.* in the North and South of Benin where increased pyrethroid resistance is also prevalent in *An. gambiae s.l.* species in South Benin ^51– 53^ than in the North, mirroring the pattern that was observed here for *An. funestus*. Resistance to DDT and permethrin is also widely distributed in *An. gambiae* in Benin ^4, 54^.

### Distribution of L119F-GSTe2 mutation in *An. funestus* populations in Benin

The high frequency of the 119F-GSTe2 resistant allele in Kpome and Doukonta where high phenotypic resistance to DDT was also observed; both results suggest that this mutation plays an important role in DDT resistance in West Africa, as previously documented ^30^. Indeed, consistent frequencies of this resistance allele were also recorded in other DDT resistant populations in Central and West Africa notably in Cameroon (52%), Ghana (44%) and Burkina Faso (25%) in accordance with the previously reported prevalence of DDT resistance in these countries ^27– 29^. The resistant 119F allele was detected in *An. funestus* populations from Tanongou, but with a relatively low frequency (35%), reflecting the moderate level of DDT resistance recorded. This result is in line with the detection of low frequencies of this resistant allele in the eastern African *An. funestus* of Uganda (20.4%) and Kenya (7.8%), which is associated with a moderate level of DDT phenotypic resistance observed in this region ^19, 26^. However, this observation is different in southern Africa where this mutation is completely absent despite recent reports of DDT resistance ^25^, suggesting that DDT resistance in southern Africa is driven by a different mechanism to that observed in West and Central Africa. These heterogeneities in L119F frequencies suggest that there are different mechanisms responsible for the DDT resistance in *An. funestus* populations across Africa.

## Conclusion

This study has generated key relevant information on the bionomics of *An. funestus* in Benin, including its seasonal distribution in a South-North transect, its *Plasmodium* infection rate and its resistance profiles to permethrin and DDT in the southern and northern ecological zones. The contrasting profiles observed between southern and northern populations of *An. funestus* were evident in the present study in terms of density, contribution to malaria transmission and resistance to insecticides. The factors behind these differences need further investigation. Overall, the high density of *An. funestus* in the south and northwestern Benin coupled with the consistent high *Plasmodium* infection level of this *Anopheles* species and its high resistance to insecticides in the South strengthens the need for more research on this species for improved performances of malaria control programs in Benin.

## Data availability

Raw data are available at the Open Science Framework: DOI, 10.17605/OSF.IO/Y3B8P ^55^.

## Abbreviations

INSAE: Institut National de la Statistique et de l'Analyse Economique; DDT: Dichlorodiphenyltrichloroethane; m/r: mosquito per room; *spp:* Species; PCR: Polymerase Chain Reaction; WHO: World Health Organization.

## Supplementary material

**Supplementary Table 1:** The surveyed 46 localities, and the species of mosquitoes collected in each locality.
