# Supplementary material for: Mapping the distribution of Anopheles funestus across Benin highlights a sharp contrast of susceptibility to insecticides and infection rate to Plasmodium between southern and northern populations
Source: Wellcome Open Res. 2017 Mar 3;1:28. Originally published 2016 Dec 14. [Version 2] doi: 10.12688/wellcomeopenres.10213.2 (PMC5300096; doi:10.12688/wellcomeopenres.10213.2)
Supplement: Supplementary file 1 [file wellcomeopenres-1-11888-s0000.tgz › 800584ac-4461-4d91-9da3-e02252145bb2.docx]

**Table S1 :** The surveyed 46 localities, and the species of mosquitoes collected in each locality.

| **N°** | **Localities** | **Climatic/ecology** | ***An. gambiae*** | ***An. funestus*** | ***Culex*** | ***Aedes*** | ***Mansonia*** | **Total** |
| --- | --- | --- | --- | --- | --- | --- | --- | --- |
| 1 | Bodjecali | Dry-Sudanese region | 8 | 0 | 3 | 0 | 0 | 11 |
| 2 | Borone | Sub-Sudanese region | 0 | 0 | 12 | 0 | 0 | 12 |
| 3 | Hêtin | Sub-equatorial region | 735 | 0 | 6 | 0 | 0 | 741 |
| 4 | Ilema (Dassa) | Sub-equatorial region | 29 | 0 | 0 | 0 | 0 | 29 |
| 5 | Kpinnou | Sub-equatorial region | 3 | 0 | 22 | 0 | 0 | 25 |
| 6 | Madecali | Dry-Sudanese region | 13 | 0 | 4 | 0 | 0 | 17 |
| 7 | Malanville centre | Dry-Sudanese region | 28 | 0 | 9 | 0 | 0 | 37 |
| 8 | Agonssa | Sub-equatorial region | 13 | 0 | 28 | 0 | 0 | 41 |
| 9 | Azili | Sub-equatorial region | 26 | 0 | 0 | 0 | 0 | 26 |
| 10 | Bamè | Sub-equatorial region | 8 | 0 | 0 | 0 | 0 | 8 |
| 11 | Boukoumbé | Wet-Sudanese region | 15 | 0 | 0 | 0 | 0 | 15 |
| 12 | Cobly- Nouagou | Wet-Sudanese region | 3 | 0 | 2 | 0 | 0 | 5 |
| 13 | Cobly-Zongo | Wet-Sudanese region | 2 | 0 | 4 | 0 | 0 | 6 |
| 14 | Coutagou | Wet-Sudanese region | 4 | 0 | 3 | 0 | 0 | 7 |
| 15 | Djetokpa | Sub-equatorial region | 11 | 0 | 3 | 0 | 0 | 14 |
| 16 | Jambala Kodilou | Wet-Sudanese region | 14 | 0 | 3 | 0 | 0 | 17 |
| 17 | Kpoto | Sub-equatorial region | 19 | 0 | 0 | 0 | 0 | 19 |
| 18 | Tchawasaga | Wet-Sudanese region | 5 | 0 | 3 | 0 | 0 | 8 |
| 19 | Faba (Angaradebou) | Dry-Sudanese region | 3 | 0 | 0 | 0 | 0 | 3 |
| 20 | Camp peulh Gome(Glazoué) | Sub-Sudanese region | 6 | 0 | 0 | 0 | 0 | 6 |
| 21 | Sowe (Glazoué) | Sub-Sudanese region | 8 | 0 | 0 | 0 | 0 | 8 |
| 22 | Sonsoro (Kandi) | Dry-Sudanese region | 13 | 0 | 0 | 0 | 0 | 13 |
| 23 | Sebou | Sub-Sudanese region | 8 | 0 | 1 | 0 | 0 | 9 |
| 24 | Thian | Sub-Sudanese region | 11 | 0 | 6 | 0 | 0 | 17 |
| 25 | Aladji-Kpara | Sub-Sudanese region | 13 | 0 | 1 | 0 | 0 | 14 |
| 26 | Kpekikinou | Sub-Sudanese region | 24 | 0 | 1 | 0 | 0 | 25 |
| 27 | Mounin (Malanville) | Dry-Sudanese region | 33 | 0 | 7 | 0 | 0 | 40 |
| 28 | Saah (Kandi) | Dry-Sudanese region | 9 | 0 | 0 | 0 | 0 | 9 |
| 29 | Tissarou (Kandi) | Dry-Sudanese region | 14 | 0 | 0 | 0 | 0 | 14 |
| 30 | Namarou (Kandi) | Dry-Sudanese region | 39 | 0 | 0 | 0 | 0 | 39 |
| 31 | Congou (Kandi) | Dry-Sudanese region | 1 | 0 | 0 | 0 | 0 | 1 |
| 32 | Banikani (Kandi) | Dry-Sudanese region | 2 | 0 | 0 | 0 | 0 | 2 |
| 33 | Tohonou | Sub-equatorial region | 2 | 0 | 0 | 0 | 0 | 2 |
| 34 | Togbadji | Sub-equatorial region | 9 | 0 | 0 | 0 | 0 | 9 |
| 35 | Doukonta- adjhikomè | Sub-equatorial region | 4 | 0 | 9 | 0 | 0 | 13 |
| 36 | Dadahoue | Sub-equatorial region | 0 | 0 | 2 | 0 | 0 | 2 |
| 37 | Doukonta- allomè | Sub-equatorial region | 489 | 15 | 78 | 0 | 24 | 606 |
| 38 | Zoundji | Sub-equatorial region | 37 | 3 | 0 | 0 | 0 | 40 |
| 39 | Zougueme | Sub-equatorial region | 38 | 1 | 0 | 0 | 0 | 39 |
| 40 | Kpassa | Sub-Sudanese region | 12 | 0 | 4 | 0 | 0 | 16 |
| 41 | Kouforpissiga | Wet-Sudanese region | 8 | 3 | 0 | 0 | 0 | 11 |
| 42 | Cobly centre | Wet-Sudanese region | 0 | 1 | 0 | 0 | 0 | 1 |
| 43 | Pahou | Sub-equatorial region | 18 | 57 | 131 | 0 | 0 | 206 |
| 44 | Tanongou | Wet-Sudanese region | 21 | 229 | 4 | 3 | 0 | 257 |
| 45 | Akoha | Sub-Sudanese region | 0 | 0 | 8 | 0 | 0 | 8 |
| 46 | Kpome | Sub-equatorial region | 325 | 243 | 120 | 23 | 20 | 731 |
|  | **Total** | | 2083 | 552 | 474 | 26 | 44 | 3179 |
