# Supplementary material for: Mapping the distribution of Anopheles funestus across Benin highlights a sharp contrast of susceptibility to insecticides and infection rate to Plasmodium between southern and northern populations
Source: Wellcome Open Res. 2017 Mar 3;1:28. Originally published 2016 Dec 14. [Version 2] doi: 10.12688/wellcomeopenres.10213.2 (PMC5300096; doi:10.12688/wellcomeopenres.10213.2)
Supplement: Peer review history

# Peer review: Referee response for version 2

**DOI:** 10.21956/wellcomeopenres.11888.r20670

**Published:** 2017-03-07

**Authors:** Mouhamadou S. Chouaibou

**Affiliations:** 1 Centre Suisse de Recherches Scientifiques en Côte d'Ivoire, Abidjan, Cote d'Ivoire

I have read this submission. I believe that I have an appropriate level of expertise to confirm that it is of an acceptable scientific standard.

# Peer review: Referee response for version 2

**DOI:** 10.21956/wellcomeopenres.11888.r20669

**Published:** 2017-03-06

**Authors:** Mamadou Ousmane Ndiath

**Affiliations:** 1 G4 Malaria Group, Institut Pasteur of Madagascar, BP 1274, Ambatofotsikely Avaradoha, 101 Antananarivo, Madagascar

I have read this submission. I believe that I have an appropriate level of expertise to confirm that it is of an acceptable scientific standard.

# Peer review: Referee response for version 1

**DOI:** 10.21956/wellcomeopenres.11002.r19465

**Published:** 2017-01-30

**Authors:** Mamadou Ousmane Ndiath

**Affiliations:** 1 G4 Malaria Group, Institut Pasteur of Madagascar, BP 1274, Ambatofotsikely Avaradoha, 101 Antananarivo, Madagascar

In a context marked by widespread insecticide resistance in different anopheles populations, knowledge of the bionomic of malaria vector such as *An. funestus* and measured insecticide susceptibilities are prerequisite for effective vector control. Indeed, *An*. *funestus* is one of the key malaria vectors in Africa and in this respect deserves more attention. The paper by Djouaka *et al.* goes in the same direction. This is a solid manuscript and generally very clearly written; the Background is very clearly focused, and the Discussion and conclusions warranted by the data. In this, I strongly encourage the publication of this manuscript. My minor comments are below: 

**Methods**

**Plasmodium infection rate of *An. funestus* populations from surveyed localities**

 I recommend the authors to give the different sequences of the primers used ( *P. ovale, vivax, malariae* and *falciparum*).

**Insecticides susceptibility**

 I suggest the authors to develop this part by giving any information about the number of ovipositing females, the number of tested mosquitoes per molecules (DDT and permethrin) and the number of replicates, the origin of impregnated papers (from Vector Control Research Unit, University Sains Malaysia, Penang, Malaysia??), condition of test (relative humidity and temperature) and insectary conditions.

**Data analysis**

 In this section, it should be mentioned the used statistical tests

**Results**

 Specify the test used in all results

- ***Plasmodium infection rate of identified members of An. funestus group***:  (P=0.0001) what test was used? Fisher’s exact test, chi-squared test? or Pearson test?....
- Same thing to the ***Screening of L119F-GSTe2….section*** (P ⩽0.001).

**Tables and figures**

- In all Tables and figures, for clarity please add the collection dates in the title
- Figure 3: The data represent mortality mate? means? medians? with 95 % confidence intervals? Please make clear.
- Figure 4: What does f (S) and f(R) represent? Please specify in the title.

I have read this submission. I believe that I have an appropriate level of expertise to confirm that it is of an acceptable scientific standard.

# Response

**Published:** 2017-02-21

**Authors:** Rousseau Djouaka

**Affiliations:** International institute of Tropical Agriculture, Benin

**Memo : How we addressed comments from Reviewer 2**

**Methods**

**Comment 1**: *Plasmodium infection rate of An. funestus populations from surveyed localities: I recommend the authors to give the different sequences of the primers used (P. ovale, vivax, malariae and falciparum).*

 For this analysis as described in the methodology, primers and probes were used: Forward (F), PlasF (5'-GCTTAGTTACGATTAATAGGAGTAGCTTG-3') and reverse (R), PlasR (5'- GAAAATCTAAGAATTTCACCTCTGACA-3') primers. Specific probes for *plasmodium *species detection: 5'-TCTGAATACGAATGTC-3' labelled with FAM for *P. falciparum* detection and 5'-CTGAATACAAATGCC-3' labelled with HEX for *P. ovale *or *P. vivax* and *P. malariae* detection were also used.

**Insecticides susceptibility**

**Comment 2** : *I suggest the authors to develop this part by giving information about:*

  

- *the number of ovipositing females*

For Tanongou, 75 *Anopheles funestus s. s.* oviposited out of 110 that were subjected to forced-egg laying technique while for Doukonta, 9 mosquitoes oviposited out of 15 that were subjected to forced-egg laying technique.

  

- *the number of tested mosquitoes per molecules (DDT and permethrin) and the number of replicates*

 

 100 *Anopheles funestus s. s. *were tested for each insecticide with 4 and 5 replicates in Doukonta and Tanongou respectively.

  

- *the origin of impregnated papers (from Vector Control Research Unit, University Sains Malaysia, Penang, Malaysia??)*

Impregnated papers were purchased from Vector Biology Department, Liverpool School of Tropical Medicine, UK.

  

- *condition of test (relative humidity and temperature) and insectary conditions.*

 

 Insectary and Bioassay room were at a temperature of 25-27°C and relative humidity of 80±5%.

**Data analysis and Results**

**Comments 3 & 4:**

***3.** In this section, it should be mentioned the used statistical tests.*

***4. **Plasmodium infection rate of identified members of An. funestus group:  (P=0.0001) what test was used? Fisher’s exact test, chi-squared test? or Pearson test?....*

*Same thing to the Screening of L119F-GSTe2….section (P ⩽0.001).*

 Fisher’s exact test was used to determine the significant levels (P-values) of the *Plasmodium *infection rates and L119F-GSTe2 allelic frequency of *Anopheles funestus* in the South compared to the North of Benin.

**Figures and Tables**

**Comment 5:**

  

- *In all Tables and figures, for clarity please add the collection dates in the title*

This comment has been addressed in the latest version of the article. See titles of figures and tables.

  

- *Figure 3: The data represent mortality rate? means? medians? with 95 % confidence intervals? Please make clear.*

 

 The data on Figure 3 represents the mortality rates of mosquitoes subjected to insecticide susceptibility tests and error bars represent the standard deviation of the mean.

  

- *Figure 4: What does f(S) and f(R) represent? Please specify in the title.*

 

**Figure 4**: Allelic frequency of the L119F-GSTe2 mutation in wild *Anopheles funestus *populations (F0) from Kpome (South Benin), Doukonta (South Benin) and Tanongou (North Benin). f(S) represents frequency of susceptible allele and f(R) represents frequency of resistant allele in the species population.

# Peer review: Referee response for version 1

**DOI:** 10.21956/wellcomeopenres.11002.r18496

**Published:** 2016-12-19

**Authors:** Mouhamadou S. Chouaibou

**Affiliations:** 1 Centre Suisse de Recherches Scientifiques en Côte d'Ivoire, Abidjan, Cote d'Ivoire

The research question is clearly defined and the study design appropriate.

 The method section provides sufficient details to allow the repeatability of the work. In overall, the paper is scientifically good and provide relevant information on *Anopheles funestus* bionomy in Benin. However, there is one minor change required; In the Methods section, susceptibility test paragraph, line 14, the author states that : ‘The wild population of *An*. *funestus* was exposed to nontreated insecticide papers as a control due to lack of susceptible strains of *An. funestus*, ( *An. funestus* FANG)’. The author should remove the underlined sentence as from WHO guideline, it is not required to use the susceptible strain as control.

 Comment/question

 Why has the author limited only to the determination of seasonal variation and infection rate?

 The author could have estimated passive aggression by dividing the number of mosquitoes collected per room by the number of sleepers in the room and then estimate the EIR.

I have read this submission. I believe that I have an appropriate level of expertise to confirm that it is of an acceptable scientific standard.

# Response

**Published:** 2016-12-19

**Authors:** Rousseau Djouaka

**Affiliations:** International institute of Tropical Agriculture, Benin

**Comment 1: **In the Methods section, susceptibility test paragraph, line 14, the author states that  ‘The wild population of *An. funestus* was exposed to non treated insecticide papers as a control due to lack of susceptible strains of *An. funestus*, ( *An. funestus* FANG)’

 The comment has been considered; the underlined statement has been removed in the PDF version in the new version of this manuscript.

**Comment 2**: Why has the author limited only to the determination of seasonal variation and infection rate?

 The author could have estimated passive aggression by dividing the number of mosquitoes collected per room by the number of sleepers in the room and then estimate the EIR.

 These parameters were initially considered at the beginning of this study (the number of people who slept in the room). At some point, we noticed some inconsistencies in the number of sleepers provided by households. It was a bit difficult to verify this discrepancy because our mosquito sampling was done in the morning when most sleepers have left the rooms (morning collections). The best option was actually to use human landing collections for more accuracy of EIR but unfortunately this was not part of our study design hence, we limited our data to mosquito densities per room and plasmodium infection rate.
